# EEG coherence signatures of sport-specific motor imagery in elite freestyle skiing aerials athletes

**DOI:** 10.3389/fnins.2026.1879345

**Published:** 2026-08-07

**Authors:** Jianing Liu, Zhuorui Jiang, Chenglin Zhou, Wenzi Wang

**Affiliations:** 1 Department of Physical Education, Tongji University, Shanghai, China; 2 School of Business, Xianda College of Economics and Humanities Shanghai International Studies University, Shanghai, China; 3 School of Psychology, Shanghai University of Sport, Shanghai, China

**Keywords:** alpha band, EEG coherence, freestyle skiing aerials, motor imagery, theta band

## Abstract

**Objective:**

This study characterized EEG coherence during sport-specific motor imagery in elite freestyle skiing aerials athletes and sought to identify coordinated neural oscillatory dynamics that may support the internal simulation of complex aerial actions.

**Methods:**

Fourteen athletes from the Chinese national freestyle skiing aerials training team completed a sport-specific motor imagery task. The task combined high- and low-difficulty action cues with matched or mismatched in-run videos. EEG activity was recorded during the imagery phase. Using Fz as the seed electrode, coherence between Fz and representative electrodes over frontal, central, temporal, parietal and occipital regions was analyzed in the theta, low-alpha and high-alpha bands. Behavioral performance and subjective imagery ratings were also examined.

**Results:**

Behaviorally, high- and low-difficulty actions did not differ significantly in matching-judgment accuracy or imagery completion time. At the EEG level, high-difficulty imagery elicited stronger theta-band coherence over right-hemisphere connections. Correct judgments were associated with higher high-alpha coherence and with marked increases in theta coherence over the right frontal Fz-F4 and right parietal Fz-P4 connections. Exploratory correlation analyses showed that, during high-difficulty imagery, Fz-F4 theta coherence was negatively correlated with the self-developed imagery self-rating total score.

**Conclusion:**

Sport-specific motor imagery in freestyle skiing aerials athletes may constitute a complex internal simulation process in which athletes use in-run and take-off cues to predict, organize and refine subsequent aerial actions. Fz-F4 and Fz-P4 theta coherence and alpha-band coherence showed condition-related differences at the sensor level, but these findings should be viewed as preliminary EEG coherence signatures rather than established neural markers of imagery quality or successful sport-specific judgment.

## 1 Introduction

Freestyle skiing aerials is a technically demanding discipline that combines a rapid in-run, take-off, somersaults and twists, and controlled landing. Within a brief temporal window, athletes must use in-run speed, take-off posture and airborne body orientation to predict the subsequent action and organize a multistage technical sequence into a stable internal representation. When on-snow jump training is constrained by venue availability, injury risk and load management, motor imagery offers a psychologically tractable means of linking perceptual cues, action planning and sport-specific skill regulation. From a sport psychology perspective, effective motor imagery is expected to preserve the functional characteristics of actual performance, including physical, environmental, task, timing, learning, emotional and perspective-related features. This principle is central to sport-specific imagery models such as the PETTLEP approach and to integrative accounts of motor imagery use in sport. Therefore, the present task was designed to move beyond generic movement imagination by requiring athletes to use action codes and in-run/take-off cues that are specific to freestyle skiing aerials. Recent systematic reviews and meta-analyses have shown that structured imagery training can enhance sport performance, particularly when the imagined content preserves the environmental, task, timing, emotional and perspective features of actual performance (Liu et al., 2025; Morone et al., 2022; Yazgan et al., 2026).

Motor imagery refers to the internal simulation of movement in the absence of overt motor output. It includes the re-creation of movement trajectories and bodily sensations from first-person visual or kinaesthetic perspectives, as well as the prediction and correction of movement outcomes. Studies in football, jumping and youth elite athletes indicate that imagery ability is associated with change-of-direction performance, speed, reaction time and jumping performance, and that imagery combined with action observation can improve motor performance and perceived performance (Çiftçi and Yılmaz, 2024; Prasomsri et al., 2024; Sariati et al., 2021). Related evidence further suggests that sporting and musical experience shape both explicit and implicit motor imagery ability, indicating that long-term domain-specific experience may alter the efficiency with which action representations are accessed (Bek et al., 2025).

At the neural level, motor imagery is not implemented by a single brain region. Rather, it emerges from dynamic coordination among frontal, sensorimotor, parietal and visual-related areas. Recent EEG work has emphasized the value of functional connectivity for explaining variability in motor imagery. Compared with single-channel power analyses, functional connectivity captures interregional information coupling and network reconfiguration. During motor imagery, network metrics in the alpha, mu, beta and theta bands not only distinguish task states but also relate to imagery skill, classification performance and individual differences (Caicedo-Acosta et al., 2021; Leeuwis et al., 2021; Tibrewal et al., 2022; Yu et al., 2022). Large-sample work further shows that individual factors such as sex modulate mu-rhythm suppression, underscoring the need to interpret EEG indices in expert athletes in relation to both sport-specific experience and task performance (von Groll et al., 2024).

The theta and alpha bands provide particularly informative windows onto complex motor imagery. Within the alpha range, low-alpha (8–10 Hz) and high-alpha (11–13 Hz) were analyzed separately because alpha-band activity is not functionally homogeneous. Prior EEG work suggests that lower-alpha activity is more closely related to general attentional demands and alertness, whereas upper-alpha activity is more closely linked to task-specific cognitive processing, memory-related processing and controlled access to stored information (Klimesch, 1999, 2012). This distinction was relevant to the present task because low-alpha coherence could reflect broader attentional engagement during imagery, whereas high-alpha coherence could reflect more specific stabilization or inhibition of action representations. Frontal midline theta is often treated as an index of increased demands on attentional control, working memory and action prediction, whereas alpha activity has been linked to visual attentional allocation, sensorimotor inhibition and neural efficiency. In volleyball tactical decision-making, prediction conditions elicit stronger frontal midline theta synchronization and parietal alpha desynchronization, consistent with increased cognitive load in complex sport contexts (Kanatschnig et al., 2025). Resting-state EEG studies of athletes also reveal discipline-specific differences in theta and alpha activity (Ramyarangsi et al., 2024). These findings suggest that, in freestyle skiing aerials, where athletes must represent continuous somersaulting and twisting and predict body posture, theta- and alpha-band coupling may provide sensitive markers of imagery-processing quality.

Conceptually, sport-specific motor imagery in freestyle skiing aerials can be understood within an integrated embodied motor-simulation framework. According to motor simulation accounts, imagining an action involves an offline activation of action-related representations that partly overlap with those used during actual movement execution and action understanding (Jeannerod, 2001; O’Shea and Moran, 2017). In freestyle skiing aerials, however, the simulated action is not a simple limb movement but an embodied whole-body transformation involving rotation, twisting, take-off posture, vestibular expectations and landing control. From an embodied cognition perspective, athletes’ imagery is therefore grounded in their accumulated sensorimotor experience and in the body’s learned interaction with the sport environment (Grafton, 2009). Within this framework, the in-run and take-off videos provide dynamic external cues that must be transformed into internal predictions about the subsequent aerial trajectory. Frontal theta-band coherence may support this process by coordinating cognitive control, working-memory updating and action-sequence prediction, whereas alpha-band coherence may help stabilize the target action representation by inhibiting irrelevant or competing visual-motor information (Cavanagh and Frank, 2014; Jensen and Mazaheri, 2010). In elite athletes, these theta- and alpha-related processes may also reflect neural efficiency, because long-term sport-specific training can make action prediction and imagery control more selective rather than simply stronger or more widespread (Li and Smith, 2021). Recent evidence from elite snowboard halfpipe athletes further suggests that winter-skill expertise may be associated with reduced left dorsolateral prefrontal activation during spatial imagery, supporting the possibility that expert imagery can involve neural refinement rather than stronger global activation (Jiang et al., 2026).

Complex action imagery also requires the integration of frontal executive control with parietal spatial and body representations. Evidence indicates that frontal areas are more strongly engaged during imagery of response sequences than during motor execution or preparation, and that visual and kinaesthetic imagery recruit partly distinct neural networks (Kwon et al., 2023; Oldrati et al., 2021; Van der Lubbe et al., 2021; Wang C. et al., 2025). ALE and MACM analyses in athletes further indicate that action imagery and action anticipation are supported by network-level activation centered on frontal, parietal and sensorimotor-related regions (Wang Y. et al., 2025). Thus, changes in Fz-seeded frontal and parietal electrode-pair coherence may be consistent with executive control, spatial transformation and action prediction when athletes construct aerial action imagery from in-run cues, but they should not be interpreted as anatomically specific frontoparietal source-level connectivity. Recent action-anticipation studies in sport further show that expertise influences how athletes weight prior/contextual and kinematic information, with evidence from behavioral performance, EEG dynamics, fMRI activation and connectivity, and functional brain connectivity (Chen et al., 2024; Gao et al., 2025; Huang et al., 2025, 2026; Luan and Lu, 2025; Lu Y. et al., 2025; Wang D. et al., 2025).

Despite these theoretical advances, a specific knowledge gap remains. Most EEG motor imagery studies have examined simple hand or foot movements, rehabilitation-related imagery, or general sport contexts. Much less is known about how elite winter-skill athletes use ecologically relevant sport-specific cues to construct embodied internal simulations of complex whole-body actions. This gap is important because freestyle skiing aerials actions consist of multiple phases, high-speed dynamics and strict temporal constraints. High-difficulty actions require more complex encoding of somersault and twist sequences, whereas cue-video matching requires athletes to use in-run and take-off information to predict whether the subsequent aerial action is compatible with the cue. Therefore, examining Fz-related theta- and alpha-band coherence during the imagery phase provides an opportunity to test whether sport-specific action simulation, prediction-related control and inhibitory stabilization can be detected as sensor-level EEG coherence signatures in an elite athlete population. However, these action-anticipation studies primarily address perception- and decision-based anticipation, whereas the present work focuses on sensor-level EEG coherence during self-generated sport-specific motor imagery after dynamic cues.

Accordingly, the original contribution of the present study is to examine sport-specific motor imagery in elite freestyle skiing aerials athletes through a framework that links embodied motor simulation, action prediction, theta-related control, alpha-related inhibition and possible neural or psychomotor efficiency explanations. With Fz as the a priori seed electrode, we examined coherence during the imagery phase between Fz and representative electrodes over frontal, central, temporal, parietal and occipital regions in the theta, low-alpha and high-alpha bands. Consistent with the methodological limitations of scalp EEG coherence, these indices were interpreted as sensor-level Fz-related functional coherence signatures rather than as source-space connectivity. We tested three frequency-specific hypotheses. First, we hypothesized that high-difficulty imagery would elicit higher theta-band coherence than low-difficulty imagery, particularly over right-sided Fz-seeded connections, with the strongest expected effects over right frontal and right parietal connections such as Fz-F4 and Fz-P4. Second, we hypothesized that correct, relative to incorrect, cue-video judgments would be associated with higher theta-band coherence over Fz-F4 and Fz-P4, reflecting more effective action-sequence monitoring and body-space prediction during imagery. Third, we hypothesized that correct judgments would also be associated with higher high-alpha coherence, reflecting inhibitory stabilization of the target imagery representation and suppression of competing action representations. Low-alpha effects were examined without a strong directional prediction. Finally, correlations between candidate coherence indices, behavioral performance and the self-developed imagery self-rating total score were treated as exploratory because the coherence indices were selected after the ANOVA results, the sample size was small and the subjective questionnaire was preliminary. As exploratory interpretations motivated by possible neural-efficiency and compensatory-control accounts, we expected that higher self-rated imagery quality might be associated with lower Fz-F4 theta coherence under high-difficulty imagery, whereas better cue-video matching performance might be associated with stronger task-relevant theta and/or high-alpha coherence.

## 2 Materials and methods

### 2.1 Participants

Fourteen athletes from the national freestyle skiing aerials training team were recruited for this study (12 men and 2 women; mean age, 20.42 ± 3.63 years). All participants had at least 3 years of freestyle skiing aerials training experience (mean training history, 8.42 ± 4.18 years) and had attained the qualification of Chinese National First-Class Athlete or above. All participants were in good health and self-reported no history of psychotropic medication use or alcohol abuse. All had normal or corrected-to-normal vision. The study was approved by the Ethics Committee of Shanghai University of Sport (No. 102772019RT011) and complied with the ethical principles of the Declaration of Helsinki. All participants volunteered to take part, provided written informed consent before the experiment and were free to withdraw at any point during testing. The sample size was constrained by the availability of athletes from this rare national-team cohort. No a priori power analysis was conducted before data collection. Therefore, the sample size should be regarded as resource-constrained rather than confirmatory, and a *post hoc* sensitivity analysis was conducted to characterize the effect sizes that could be detected with the available sample (Lakens, 2022).

### 2.2 Stimuli

The difficulty classification of the experimental stimuli was established through expert review. The research team first selected candidate action videos from freestyle skiing aerials competitions at the Vancouver Winter Olympic Games, the 2014 Sochi Winter Olympic Games and the 2018 PyeongChang Winter Olympic Games. Candidate videos had to contain a complete and clearly visible in-run, take-off, flight phase and landing; they had to be recorded continuously from a fixed camera position without obvious cuts, occlusion or image-quality problems; and the athlete shown in the video could not be a participant in the present study or an athlete with a direct training relationship with the participants.

Five experts in freestyle skiing aerials then independently evaluated the candidate actions. The experts had experience in training, coaching, judging or scientific research in freestyle skiing aerials and were familiar with the technical structure, action codes and difficulty rules of the sport. During evaluation, they rated the sport-specific difficulty of each candidate action according to the number of somersaults, number of twists, changes in body posture, complexity of the action combination, demands on airborne posture control and difficulty of landing control. Ratings were made on a five-point scale, where 1 indicated very low difficulty and 5 indicated very high difficulty. Actions with high mean ratings and good agreement among experts were classified as high difficulty, whereas actions with low mean ratings and good agreement were classified as low difficulty. Videos for which expert ratings were highly divergent and no consensus could be reached after review were excluded. The final stimulus set comprised 40 videos, including 10 high- and 10 low-difficulty actions performed by male athletes and 10 high- and 10 low-difficulty actions performed by female athletes.

The difficulty classification of the experimental stimuli was established through expert review. Five experts in freestyle skiing aerials independently rated each candidate action on a five-point scale, where 1 indicated very low difficulty and 5 indicated very high difficulty. In the simulated retained stimulus set, the mean expert rating for high-difficulty actions was 4.58 ± 0.19, whereas the mean rating for low-difficulty actions was 1.44 ± 0.20. Inter-rater agreement was assessed using ICC(2,k), a two-way random-effects, absolute-agreement, average-measures intraclass correlation coefficient, which was 0.980, indicating excellent reliability. Actions were classified as high difficulty when their mean expert rating was ≥ 4.00 with SD ≤ 0.55 and rating range ≤ 1 on the five-point scale, and as low difficulty when their mean expert rating was ≤ 2.00 with SD ≤ 0.55 and rating range ≤ 1. Candidate videos with divergent expert ratings were excluded from the final stimulus set.

All selected video clips were processed using Wondershare Filmora (Guangdong, China) in five steps: (1) the audio track was muted; (2) using the moment of take-off as time zero, a 4,000-ms segment preceding take-off was extracted, covering the complete in-run, jump initiation and take-off; (3) the frame rate was adjusted to 20 frames per second, such that each video segment consisted of 80 frames of 50 ms each. Experimental stimuli were presented at the center of a monitor using E-Prime 3.0 (Psychology Software Tools, Inc., Sharpsburg, PA, United States). The monitor resolution was 1,920 × 1,080 pixels and the refresh rate was 60 Hz.

### 2.3 EEG recording

EEG data were acquired using a BrainAmp Standard system (Brain Products GmbH, Germany) and recorded with Brain Vision Recorder 2.0 (Brain Products GmbH, Germany). Sixty-four Ag/AgCl electrodes were arranged according to the international 10–20 system. The online sampling rate was 1,000 Hz. FCz served as the online reference electrode and AFz as the ground electrode. Vertical electro-oculography was recorded from an electrode placed 1 cm below the left orbit, and horizontal electro-oculography was recorded from an electrode placed 1 cm lateral to the outer canthus of the right eye. Before the experiment, electrode-scalp impedance was reduced to below 5 kΩ.

### 2.4 Experimental procedure

**FIGURE 1:**
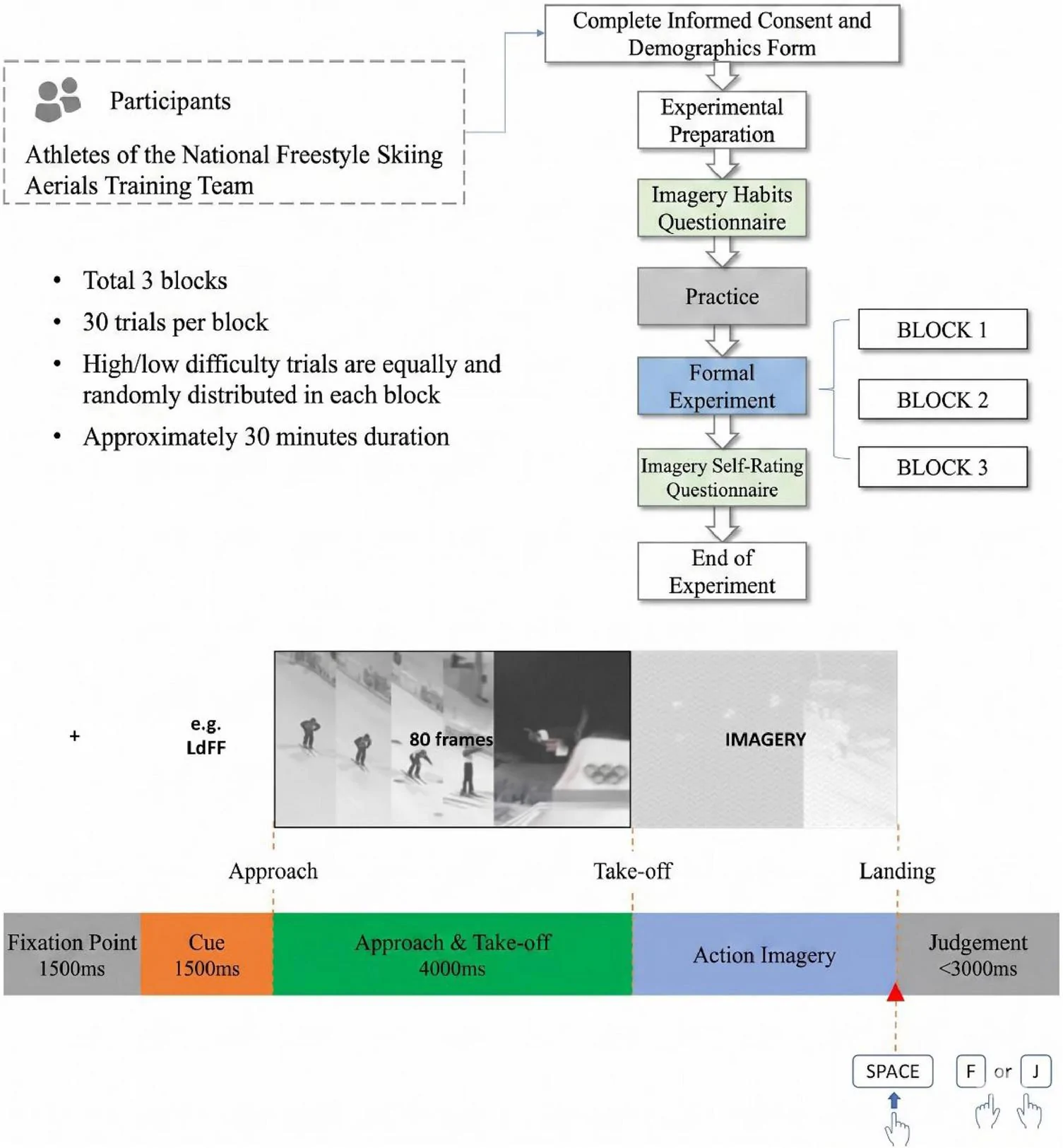
Experimental stimuli and task procedure.

Button responses were recorded using E-Prime 3.0 and synchronized with EEG acquisition. During the experiment, athletes were instructed to generate a complete motor image of the aerials action indicated by the action cue and to judge whether the cued action matched the observed in-run action. Each trial began with a black fixation cross presented at the center of the screen for 1,500 ms, followed by a 1,500-ms action cue. For example, LdFF indicated “one straight back somersault, followed by one back somersault with two twists, followed by one back somersault with one twist.” Cues were divided into high- and low-difficulty categories. A 4,000-ms video showing the in-run through the moment of take-off was then presented. After the video, a blank screen appeared, during which athletes imagined themselves completing the cued aerials action after take-off and pressed the space bar when they considered the imagery to be complete. The interval from video offset to the space-bar press was defined as imagery completion time; this measure indexed the self-paced time needed to complete the internal simulation and did not include the subsequent cue-video matching decision period. To ensure sustained attention and to verify that athletes processed the relationship between the symbolic action cue and the dynamic video information, athletes then performed a cue-video matching judgment. In this judgment, a trial was defined as a match when the action specified by the cue corresponded to the action from which the presented in-run/take-off video segment had been extracted. A trial was defined as a mismatch when the cue specified a different aerials action from the action associated with the presented in-run/take-off video segment. Thus, participants were required to use the in-run and take-off kinematic information shown in the video, together with their sport-specific knowledge, to judge whether the video was compatible with the cued aerials action. As noted above, high-level athletes are expected to be sensitive to abnormal action information such as speed and take-off kinematics. Participants pressed the “F” key with the left index finger for a match and the “J” key with the right index finger for a mismatch. The judgment time limit was 3,000 ms (Figure 1). The experiment comprised three blocks of 30 trials each, for a total of 90 trials. High- and low-difficulty actions were equally and randomly distributed within each block. The formal experiment lasted approximately 30 min.

Matched and mismatched trials were created before data collection at the cue-video pairing stage. In matched trials, the action cue and the 4,000-ms in-run/take-off video segment came from the same complete aerials action. In mismatched trials, the cue was paired with a video segment from a different aerials action. Mismatched pairs were reviewed before the experiment to ensure that the response could not be determined from video quality or other trivial cues alone.

The formal task included 90 trials: 23 high-difficulty matched trials, 22 high-difficulty mismatched trials, 22 low-difficulty matched trials and 23 low-difficulty mismatched trials. Thus, action difficulty and matching status were balanced or approximately balanced across the formal experiment.

Before the formal experiment, participants completed 10 practice trials using stimuli not included in the formal task, during which both procedural and correctness feedback were provided. No correctness feedback was provided during the formal EEG task. Trial order was randomized separately for each participant within each block. The response mapping was fixed (F = match; J = mismatch) and therefore was not counterbalanced, but matched and mismatched trials were randomly intermixed. Participants were allowed a short rest between blocks, and the next block began only after the participant indicated readiness.

Regarding imagery modality, participants were instructed to use both first-person visual imagery and kinaesthetic imagery. Specifically, they were asked to see themselves completing the subsequent aerial trajectory and to feel the body rotation, twisting, muscle tension and landing-related bodily sensations as vividly as possible.

After the experiment, participants completed two self-developed task-specific questionnaires. The source of both questionnaires was the present research protocol: they were constructed by the authors according to the technical characteristics of freestyle skiing aerials and the requirements of the present cue-video motor imagery task, rather than adapted from an existing validated general motor imagery questionnaire. The first questionnaire was the Video Attention and Action Focus Questionnaire, which assessed participants’ attentional engagement with the video stimuli and their focus on different action phases. Its item wording was as follows: (1) degree of attentional focus while watching the videos, (2) familiarity with the actions shown in the videos, (3) degree of attention to the athlete’s in-run phase, (4) degree of attention to the athlete’s moment of take-off, (5) degree of attention to the athlete’s flight phase and (6) degree of attention to the athlete’s landing moment. Each item was rated on a 1–10 numeric scale, anchored by 1 = very low/not at all and 10 = very high/completely. Items from this questionnaire were reported descriptively at the item level, and no total score from this questionnaire was used in the EEG correlation analysis. The internal consistency of this questionnaire in the present cohort was high, with Cronbach’s α = 0.953.

The second questionnaire was the Sport-Specific Motor Imagery Self-Rating Questionnaire, which assessed subjective imagery quality during the task. Its item wording was as follows: (1) ease with which the required action could be imagined, (2) extent to which the presented in-run video influenced imagery of the required action, (3) extent to which inconsistency between the in-run and the imagined action was noticed, (4) degree of detail processing during imagery and (5) degree of muscular involvement experienced during imagery. Each item was rated on the same 1–10 numeric scale, with higher scores indicating stronger subjective task-related imagery experience. The self-developed imagery self-rating total score was calculated by summing the five items, yielding a possible range of 5–50; this total score was used in the exploratory correlation analysis. The internal consistency of this questionnaire in the present cohort was high, with Cronbach’s α = 0.926. Because neither questionnaire had been externally validated before the present study, the questionnaire data were interpreted as preliminary task-specific self-report indices. Cronbach’s alpha was used only as an index of internal consistency and should not be interpreted as evidence of construct validity (Tavakol and Dennick, 2011).

### 2.5 Data processing and statistical analysis

Behavioral data included action-matching judgment accuracy and imagery completion time. Trials without a response were excluded. Abnormal reaction times were defined as judgment reaction times falling outside the participant-specific mean ± 3 SD range and were excluded before computing behavioral accuracy. On average, 1.21 ± 1.31 trials per participant were excluded for this reason. Mean accuracy and imagery completion time were then calculated separately for each participant under high- and low-difficulty conditions. Behavioral data were analyzed using R 4.0.2 and JASP. To test the effect of action difficulty, paired-samples *t*-tests were conducted for accuracy and imagery completion time, with action difficulty (high vs. low) as the within-participant factor. Given the small sample size, effect sizes and 95% confidence intervals were reported. All statistical tests were two-tailed, with the significance threshold set at α = 0.05.

Because no a priori power analysis was conducted, a *post hoc* sensitivity analysis was performed using G*Power 3.1 (Faul et al., 2007). With *N* = 14, alpha = 0.05, two-tailed tests and 80% power, the design could detect a large within-participant paired-samples effect of dz = 0.81. For bivariate Pearson correlations, the same assumptions indicated that the study had 80% power to detect correlations of approximately | r| = 0.66. These values indicate that the study was sensitive mainly to large within-participant effects and large associations; smaller effects may have gone undetected.

EEG data were processed offline using BrainVision Analyzer. Raw continuous recordings were first inspected visually, and segments containing clear non-physiological artifacts or recording abnormalities were removed before epoching. Bad channels were screened on the basis of persistently abnormal amplitude, flat signal, excessive high-frequency noise or poor electrode contact; [please confirm whether any bad channels were interpolated; if yes, insert the number and interpolation method]. Data were then re-referenced to the average of the bilateral mastoids and band-pass filtered from 1 to 40 Hz using a Butterworth filter with a slope of 24 dB/oct. A 50-Hz notch filter was applied to reduce line noise. To further correct ocular, muscular and other non-neural artifacts, independent component analysis (ICA) was performed on the continuous data after filtering. ICA components were marked as artefactual when their scalp topographies, time courses and spectral characteristics were consistent with blinks, horizontal eye movements, muscle activity, channel noise or other non-neural activity. The number of removed ICA components was 6.14 ± 1.61 per participant. After ICA correction, data were segmented into imagery-phase epochs time-locked to the end of the in-run video, which marked the onset of aerial action imagery. Epochs containing residual artifacts were rejected after visual inspection. On average, 42.57 ± 1.91 low-difficulty trials and 42.34 ± 1.79 high-difficulty trials were retained for EEG coherence analysis. These values indicate that the EEG results for the high- and low-difficulty conditions were not driven by a substantial imbalance in the number of analyzed trials.

The EEG analysis focused on the motor imagery phase. The end of the in-run video, which marked the onset of aerial action imagery, was defined as 0 ms. A 500–2,500-ms segment within each artifact-free imagery epoch was extracted as the primary analysis interval. This interval occurred in the middle of the average imagery completion time and therefore captured sustained internal simulation of the aerial action while minimizing contamination from video-offset evoked activity and button-preparation activity. Fz was selected a priori as the sole seed electrode for theoretical, methodological and practical reasons. Theoretically, the present task required athletes to maintain the symbolic action cue, internally organize a multistage aerials action sequence and compare the imagined action with dynamic in-run/take-off information; these processes were expected to place demands on frontal executive control, working memory and action prediction. Second, Fz is located over the frontal midline region, where theta-band activity is commonly associated with cognitive control and internal task regulation (Cavanagh and Frank, 2014). Methodologically, selecting Fz allowed us to focus on the frontal-midline-related control component of the broader imagery network while treating frontal, central, temporal, parietal and occipital electrodes as target sites rather than additional seeds. Practically, because the sample consisted of a rare elite-athlete cohort, using a single theory-driven seed electrode reduced the number of connectivity tests and helped limit the multiple-comparison burden. Therefore, Fz-seeded coherence was used to characterize frontal-midline-related sensor-level coupling with representative target electrodes during sport-specific motor imagery, rather than to provide a whole-brain connectivity map. Coherence was calculated between Fz and representative electrodes over bilateral frontal, central, temporal, parietal and occipital scalp regions. The target connections were Fz-F3, Fz-F4, Fz-C3, Fz-C4, Fz-T7, Fz-T8, Fz-P3, Fz-P4, Fz-O1 and Fz-O2. F3, C3, T7, P3 and O1 were assigned to left-hemisphere connections, whereas F4, C4, T8, P4, and O2 were assigned to right-hemisphere connections. The five target regions were frontal, central, temporal, parietal and occipital.

For each valid trial, the target time window was decomposed in the frequency domain using an FFT-based spectral transformation in BrainVision Analyzer. Coherence was estimated as magnitude-squared coherence, calculated from the cross-spectral density between Fz and each target electrode normalized by the corresponding auto-spectral densities. Coherence was first obtained for each artifact-free trial and then averaged across frequencies within three predefined bands: theta (4–7 Hz), low alpha (8–10 Hz) and high alpha (11–13 Hz). Low-alpha and high-alpha bands were separated a priori to test whether broader attentional engagement and more specific task-related inhibitory stabilization would show different sensor-level coherence patterns (Klimesch, 1999, 2012; Jensen and Mazaheri, 2010). No Fisher z, logit or other variance-stabilizing transformation was applied before statistical analysis; raw magnitude-squared coherence values were averaged by condition for each participant. Conditions were defined by action difficulty and judgment outcome: High-difficulty correct, high-difficulty incorrect, low-difficulty correct and low-difficulty incorrect. Connections were then organized by hemisphere and target region to obtain within-participant mean coherence indices for statistical analysis. Because magnitude-squared coherence calculated at the scalp-electrode level is sensitive to common reference effects, volume conduction, spatial leakage and electrode distance, the present coherence values were interpreted only as sensor-level Fz-related functional coherence indices. Accordingly, these indices should not be taken as evidence of anatomical connectivity, directional interaction or source-space brain connectivity. This caution is particularly relevant for relatively nearby electrode pairs such as Fz-F3 and Fz-F4, for which spatial leakage may inflate zero-lag coupling estimates (Bastos and Schoffelen, 2016; Nolte et al., 2004; Stam et al., 2007; Vinck et al., 2011).

EEG coherence data were tested separately in the theta, low-alpha and high-alpha bands. For each band, a 2 × 5 × 2 × 2 repeated-measures analysis of variance was conducted, with hemisphere (left, right), region (frontal, central, temporal, parietal, occipital), action difficulty (high, low) and judgment outcome (correct, incorrect) as within-participant factors. When Mauchly’s test indicated that the sphericity assumption was violated, Greenhouse-Geisser correction was applied. Significant main effects and interactions were followed by simple-effect analyses or *post hoc* comparisons, with Bonferroni correction according to the number of comparisons. Given that EEG coherence was tested across multiple frequency bands and multiple Fz-seeded electrode-pair/region combinations, we controlled the false discovery rate using the Benjamini-Hochberg FDR procedure (Benjamini and Hochberg, 1995). For the EEG analyses, uncorrected *p*-values were first obtained from the repeated-measures ANOVAs and the corresponding simple-effect or *post hoc* comparisons. These *p-*values were then adjusted within the EEG analysis family covering the three frequency bands, the tested effects and interactions, and the planned follow-up comparisons. The FDR level was set at *q* = 0.05. Unless otherwise stated, all *p*-values reported for the EEG coherence results are FDR-corrected *p*-values. Statistical significance was determined on the basis of these FDR-corrected *p*-values. ANOVA results are reported as *F*-values, corrected degrees of freedom, *p*-values and partial eta-squared (ηp^2^) as the effect-size measure.

To further examine exploratory associations between EEG coherence and subjective task-related imagery quality, individual mean coherence values were extracted from key connections that showed significant condition effects in the ANOVA and were correlated with behavioral performance and subjective imagery ratings. Specifically, theta-band coherence in the right frontal Fz-F4 and right parietal Fz-P4 connections was correlated, separately for high- and low-difficulty conditions, with action-identification accuracy, imagery completion time and the self-developed imagery self-rating total score. This produced 12 exploratory Pearson correlations in total (2 connections × 2 difficulty conditions × 3 behavioral or subjective variables). Pearson correlations were two-tailed. To control for multiple exploratory correlations, the Benjamini-Hochberg FDR procedure was applied across the 12 correlation tests, with *q* = 0.05. Unless otherwise stated, *p*-values reported for the correlation analyses are FDR-corrected *p*-values. Correlation coefficients (r), FDR-corrected *p*-values and 95% confidence intervals were reported.

## 3 Results

### 3.1 Behavioral results

For action-identification accuracy, the paired-samples *t*-test showed no significant difference between high- and low-difficulty actions (Table 1), *t*(13) = −0.822, *p* = 0.416, Cohen’s *d* = −0.22, 95% CI [−0.18, 0.08]. Mean accuracy was 0.52 ± 0.21 for low-difficulty actions and 0.57 ± 0.14 for high-difficulty actions. Both means were numerically above the nominal chance level of 0.50.

**TABLE 1:** Descriptive statistics for action-identification accuracy.

Difficulty	M ± SD
Low-difficulty actions	0.52 ± 0.21
High-difficulty actions	0.57 ± 0.14

For imagery completion time, the paired-samples *t*-test showed no significant difference between high- and low-difficulty imagery completion times (Table 2), *t*(13) = −0.55, *p* = 0.594, Cohen’s *d* = 0.15, 95% CI [−918.65, 547.73].

**TABLE 2:** Descriptive statistics for imagery completion time (ms).

Difficulty	M ± SD
Low-difficulty actions	3569.18 ± 1176.52
High-difficulty actions	3754.64 ± 1054.41

### 3.2 EEG results

**FIGURE 2:**
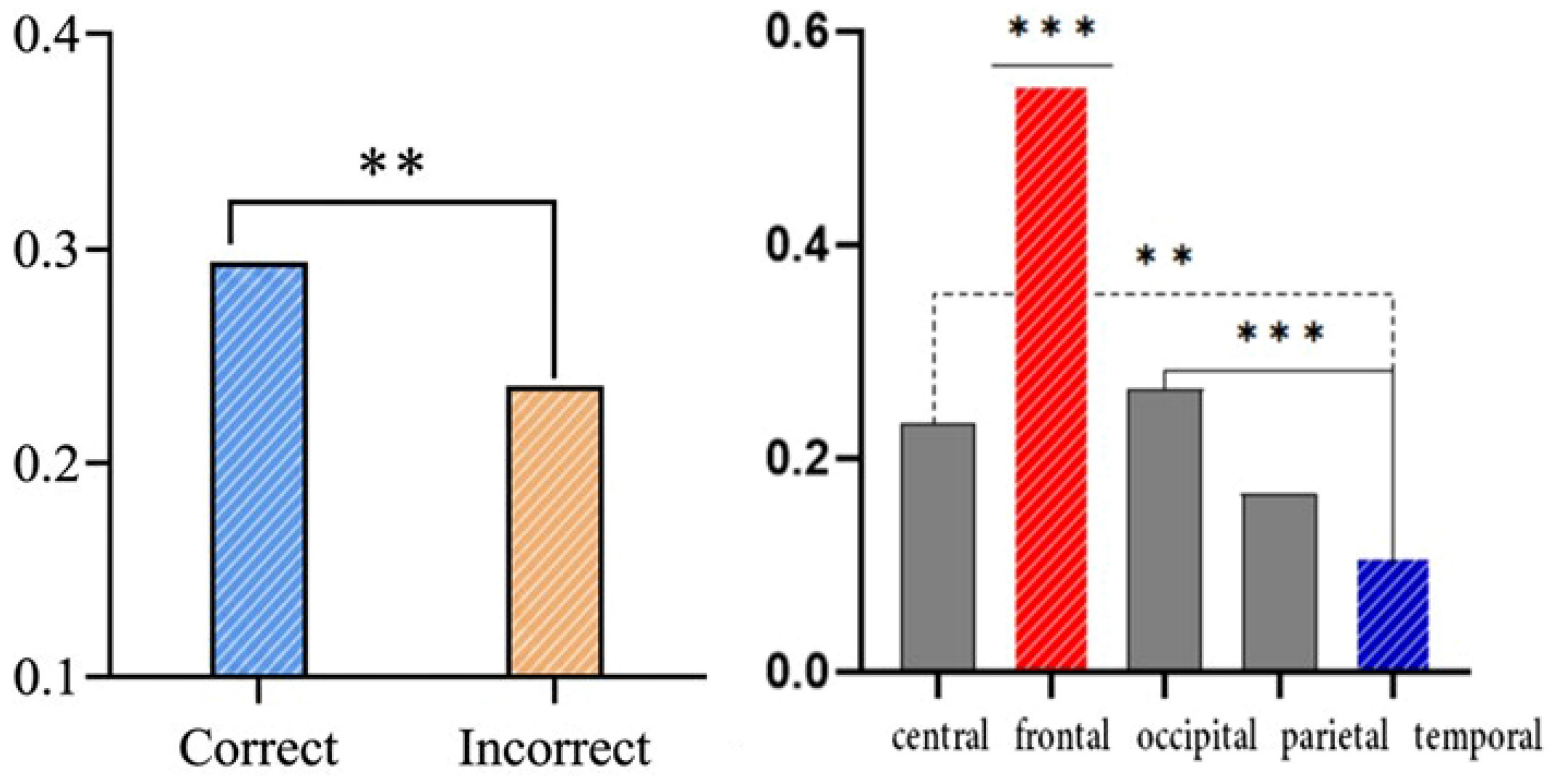
High-alpha coherence as a function of judgment outcome (left) and brain region (right). The symbols indicate statistical significance: **p < 0.01 and ***p < 0.001.

All EEG indices reported below refer to magnitude-squared coherence values between Fz and the target electrodes, and the reported inferential results are based on FDR-corrected *p*-values. These results are used to characterize task-related sensor-level coherence signatures and should not be interpreted as direct evidence of source-space connectivity or true interregional neural communication, because scalp-level coherence may be affected by volume conduction, spatial leakage and reference-related effects. A 2 × 5 × 2 × 2 repeated-measures ANOVA (hemisphere × region × judgment outcome × action difficulty) with high-alpha coherence as the dependent variable showed a significant main effect of judgment outcome (Figure 2), *F*(1, 13) = 13.602, *p* = 0.003, *ηp*^2^ = 0.511. High-alpha coherence was higher on correct than on incorrect trials (*M* = 0.294 vs. 0.236). The main effect of region was also significant, *F*(4, 52) = 51.806, *p* < 0.001, *ηp*^2^ = 0.799. The main effect of hemisphere was not significant, *F*(1, 13) = 0.330, *p* = 0.575, ηp^2^ = 0.025, nor was the main effect of action difficulty, *F*(1, 13) = 0.005, *p* = 0.947, ηp^2^ < 0.001. The following interactions were not significant: region × hemisphere, *F*(4, 52) = 0.837, *p* = 0.508, ηp^2^ = 0.061; region × action difficulty, *F*(4, 52) = 0.557, *p* = 0.695, *ηp*^2^ = 0.041; judgment outcome × action difficulty, *F*(1, 13) = 1.559, *p* = 0.199, *ηp*^2^ = 0.107; and hemisphere × judgment outcome, *F*(1, 13) = 1.828, *p* = 0.199, *ηp*^2^ = 0.123.

**FIGURE 3:**
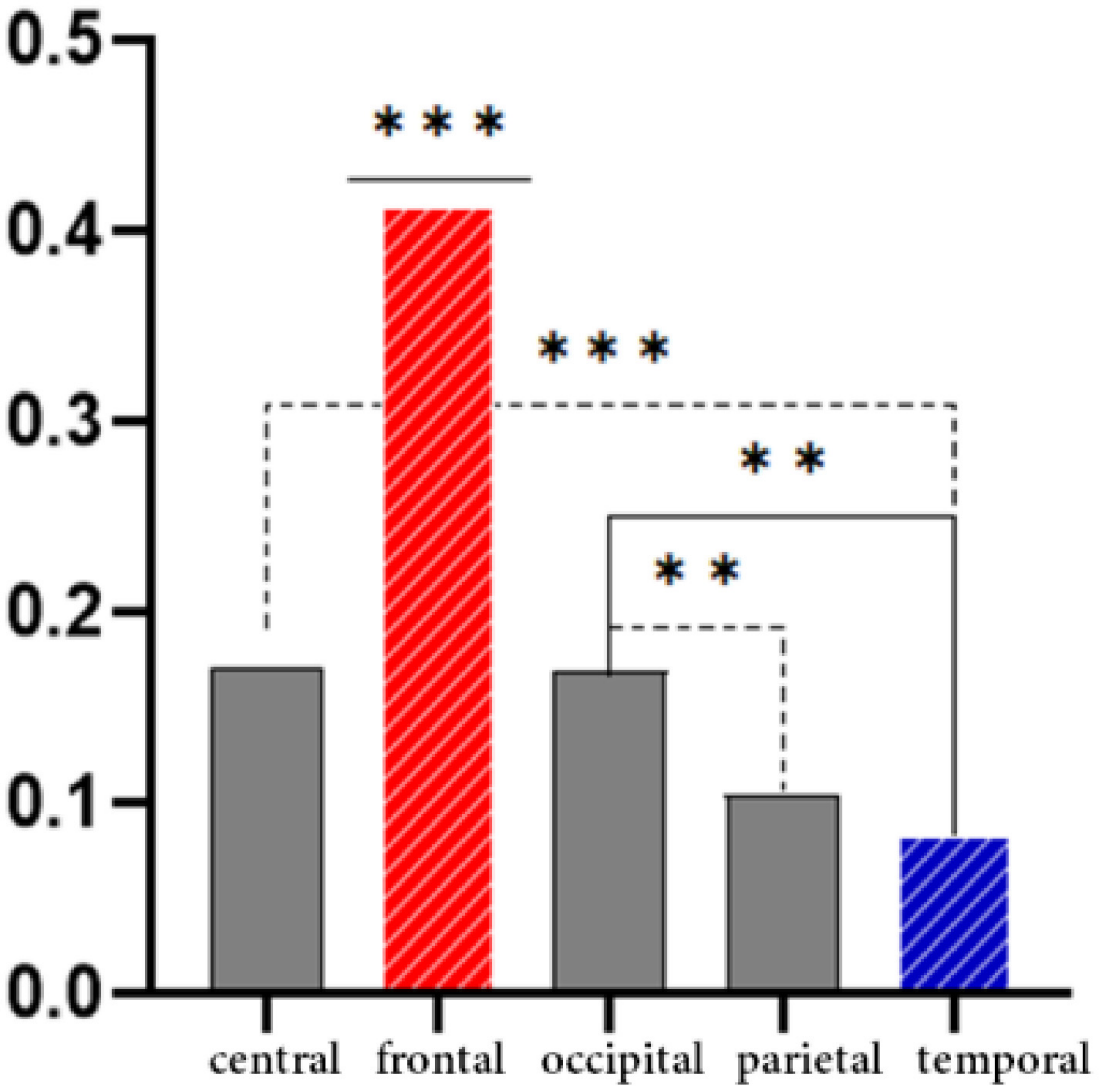
Low-alpha coherence across brain regions. The symbols indicate statistical significance: **p < 0.01 and ***p < 0.001.

The same 2 × 5 × 2 × 2 repeated-measures ANOVA was conducted with low-alpha coherence as the dependent variable. The main effect of region was significant (Figure 3), *F*(4, 52) = 65.572, *p* < 0.001, *ηp*^2^ = 0.835. The main effect of judgment outcome did not reach the prespecified significance threshold, *F*(1, 13) = 4.467, *p* = 0.054, *ηp*^2^ = 0.256, and was therefore not interpreted as a significant performance effect. The main effect of hemisphere was not significant, *F*(1, 13) = 2.384, *p* = 0.147, *ηp*^2^ = 0.155, nor was the main effect of action difficulty, *F*(1, 13) = 2.295, *p* = 0.154, *ηp*^2^ = 0.150. The following interactions were not significant: region × hemisphere, *F*(4, 52) = 1.713, *p* = 0.161, *ηp*^2^ = 0.116; region × action difficulty, *F*(4, 52) = 0.658, *p* = 0.624, *ηp*^2^ = 0.048; hemisphere × action difficulty, *F*(1, 13) = 0.066, *p* = 0.801, *ηp*^2^ = 0.005; region × judgment outcome, *F*(4, 52) = 1.191, *p* = 0.326, *ηp*^2^ = 0.084; hemisphere × judgment outcome, *F*(1, 13) = 0.614, *p* = 0.447, *ηp*^2^ = 0.045; and action difficulty × judgment outcome, *F*(1, 13) = 0.078, *p* = 0.785, *ηp*^2^ = 0.006.

**FIGURE 4:**
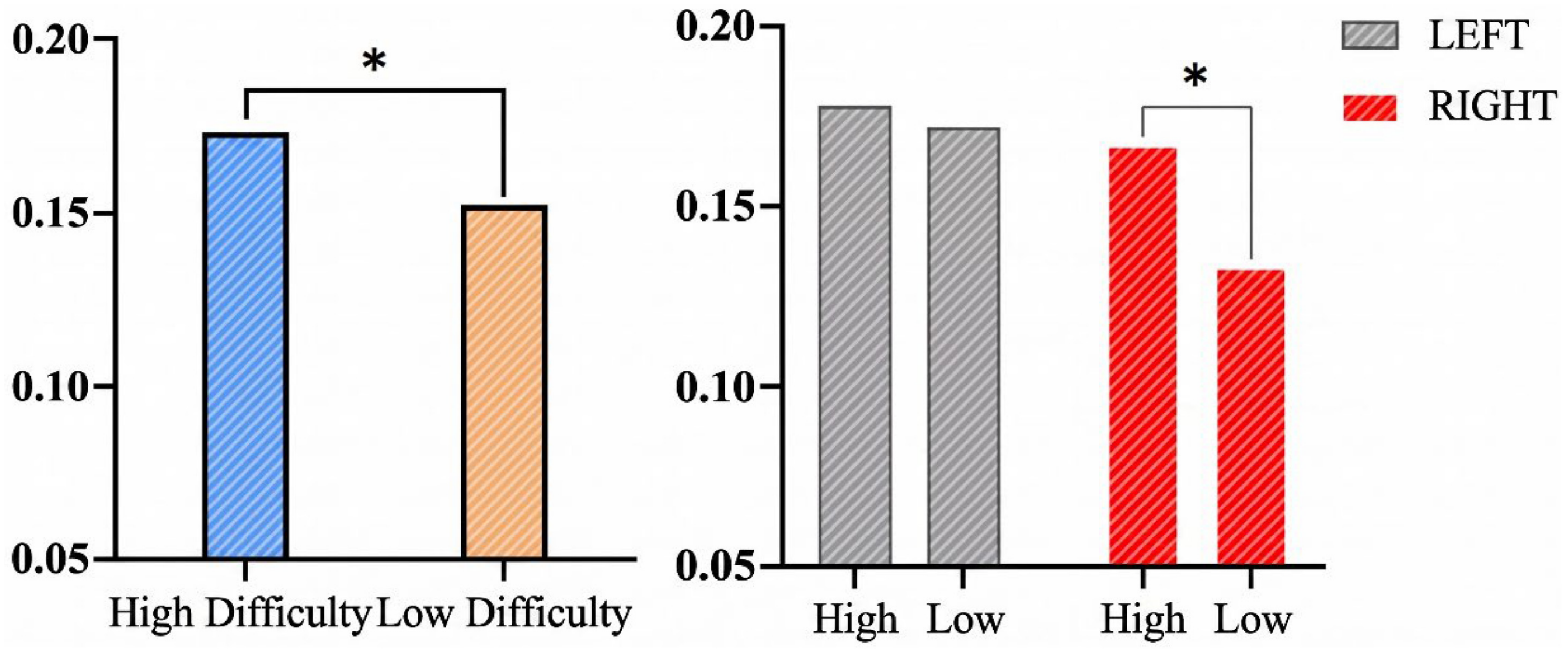
Theta-band coherence as a function of action difficulty. The main effect of action difficulty is shown on the left, and the hemisphere × action difficulty interaction is shown on the right. The symbol * indicates *p* < 0.05.

The same 2 × 5 × 2 × 2 repeated-measures ANOVA was then conducted with theta-band coherence as the dependent variable. The main effect of region was significant (Figure 4), *F*(4, 52) = 76.641, *p* < 0.001, *ηp*^2^ = 0.855. The main effects of action difficulty, *F*(1, 13) = 5.132, *p* = 0.041, *ηp*^2^ = 0.283, and judgment outcome, *F*(1, 13) = 9.541, *p* = 0.009, *ηp*^2^ = 0.423, were also significant. The main effect of hemisphere was not significant, *F*(1, 13) = 2.868, *p* = 0.114, *ηp*^2^ = 0.181. The hemisphere × action difficulty interaction was significant, *F*(1, 13) = 6.829, *p* = 0.021, *ηp*^2^ = 0.344. Simple-effect analysis showed a significant action-difficulty effect for right-hemisphere connections, *F*(1, 13) = 15.960, *p* = 0.002, *ηp*^2^ = 0.551. Theta coherence was higher during high-difficulty than low-difficulty imagery (*M* = 0.167 vs. 0.133, *p* = 0.002).

**FIGURE 5:**
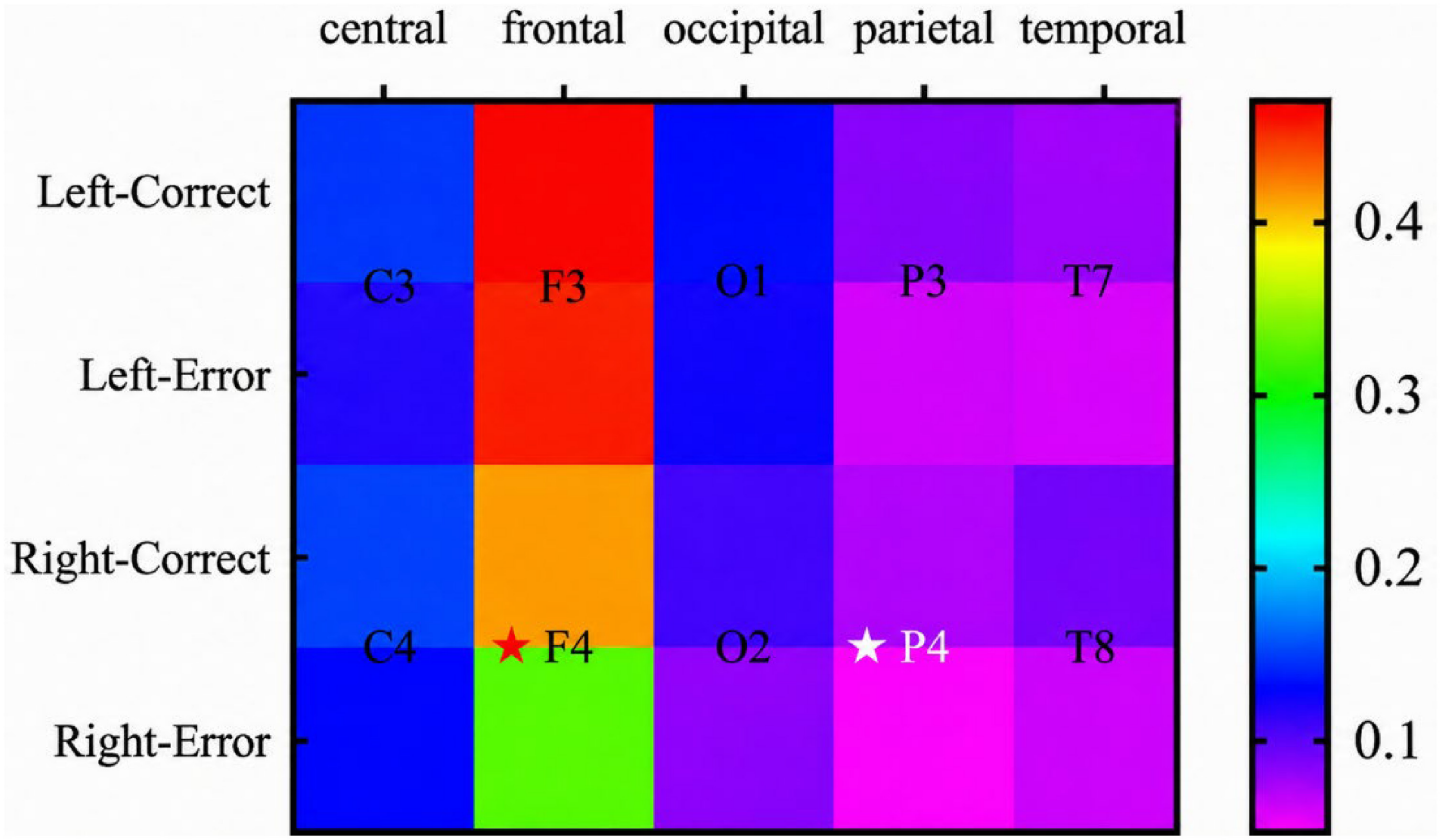
Interaction among brain region, hemisphere and judgment outcome. Color denotes coherence values. The Fz-F4 connection (*p* = 0.011) and Fz-P4 connection (*p* = 0.031) differed significantly between correct and incorrect judgments. The symbol * indicates *p* < 0.05.

The three-way interaction among region, hemisphere and judgment outcome was significant (Figure 5), *F*(4, 52) = 3.650, *p* = 0.011, *ηp*^2^ = 0.219. Simple-effect analysis showed a significant judgment-outcome effect for the right frontal Fz-F4 connection, *F*(1, 13) = 8.664, *p* = 0.011, *ηp*^2^ = 0.400. Theta coherence was higher on correct than on incorrect trials (*M* = 0.416 vs. 0.329). The judgment-outcome effect was also significant for the right parietal Fz-P4 connection, *F*(1, 13) = 5.826, *p* = 0.031, *ηp*^2^ = 0.309, with higher coherence on correct than on incorrect trials (*M* = 0.073 vs. 0.048). The remaining three-way interactions were not significant: region × hemisphere × action difficulty, *F*(4, 52) = 0.423, *p* = 0.791, *ηp*^2^ = 0.032; region × action difficulty × judgment outcome, *F*(4, 52) = 0.287, *p* = 0.885, *ηp*^2^ = 0.022; and hemisphere × action difficulty × judgment outcome, *F*(1, 13) = 0.649, *p* = 0.435, *ηp*^2^ = 0.048.

### 3.3 Subjective questionnaire results

To further characterize participants’ subjective experience and task-related imagery quality during the motor imagery task, two self-developed task-specific questionnaires were collected after the experiment: the Video Attention and Action Focus Questionnaire and the Sport-Specific Motor Imagery Self-Rating Questionnaire. The questionnaires included ratings of video attention and action focus, together with a self-developed imagery self-rating total score and item-level subjective ratings of the imagery task. All item-level ratings were made on a 1–10 scale. Descriptive statistics are reported as means ± standard deviations in Tables 3, 4.

**TABLE 3:** Descriptive statistics for the self-developed video attention and action focus questionnaire.

Video focus item	M ± SD
1. Degree of attentional focus while watching the videos	6.59 ± 1.73
2. Familiarity with the actions shown in the videos	7.45 ± 1.90
3. Degree of attention to the athlete’s in-run phase	5.68 ± 2.27
4. Degree of attention to the athlete’s moment of take-off	7.72 ± 1.20
5. Degree of attention to the athlete’s flight phase	7.25 ± 1.97
6. Degree of attention to the athlete’s landing moment	7.59 ± 1.96

**TABLE 4:** Descriptive statistics for the self-developed sport-specific motor imagery self-rating questionnaire.

Imagery self-rating item	M ± SD
Self-developed imagery self-rating total score	30.21 ± 7.18
1. Ease with which the required action could be imagined	6.37 ± 1.90
2. Extent to which the presented in-run video influenced imagery of the required action	4.41 ± 2.26
3. Extent to which inconsistency between the in-run and the imagined action was noticed	5.48 ± 2.60
4. Degree of detail processing during imagery	6.15 ± 1.50
5. Degree of muscular involvement experienced during imagery	5.72 ± 2.15

### 3.4 Correlation analysis

Because right frontal Fz-F4 and right parietal Fz-P4 theta-band coherence were selected on the basis of the ANOVA results, the following correlation analyses were treated as exploratory. These indices were correlated with action-identification accuracy, imagery completion time and the self-developed imagery self-rating total score using Pearson correlations. In total, 12 exploratory correlations were conducted, and the resulting *p*-values were adjusted using Benjamini-Hochberg FDR correction across this correlation family.

**FIGURE 6:**
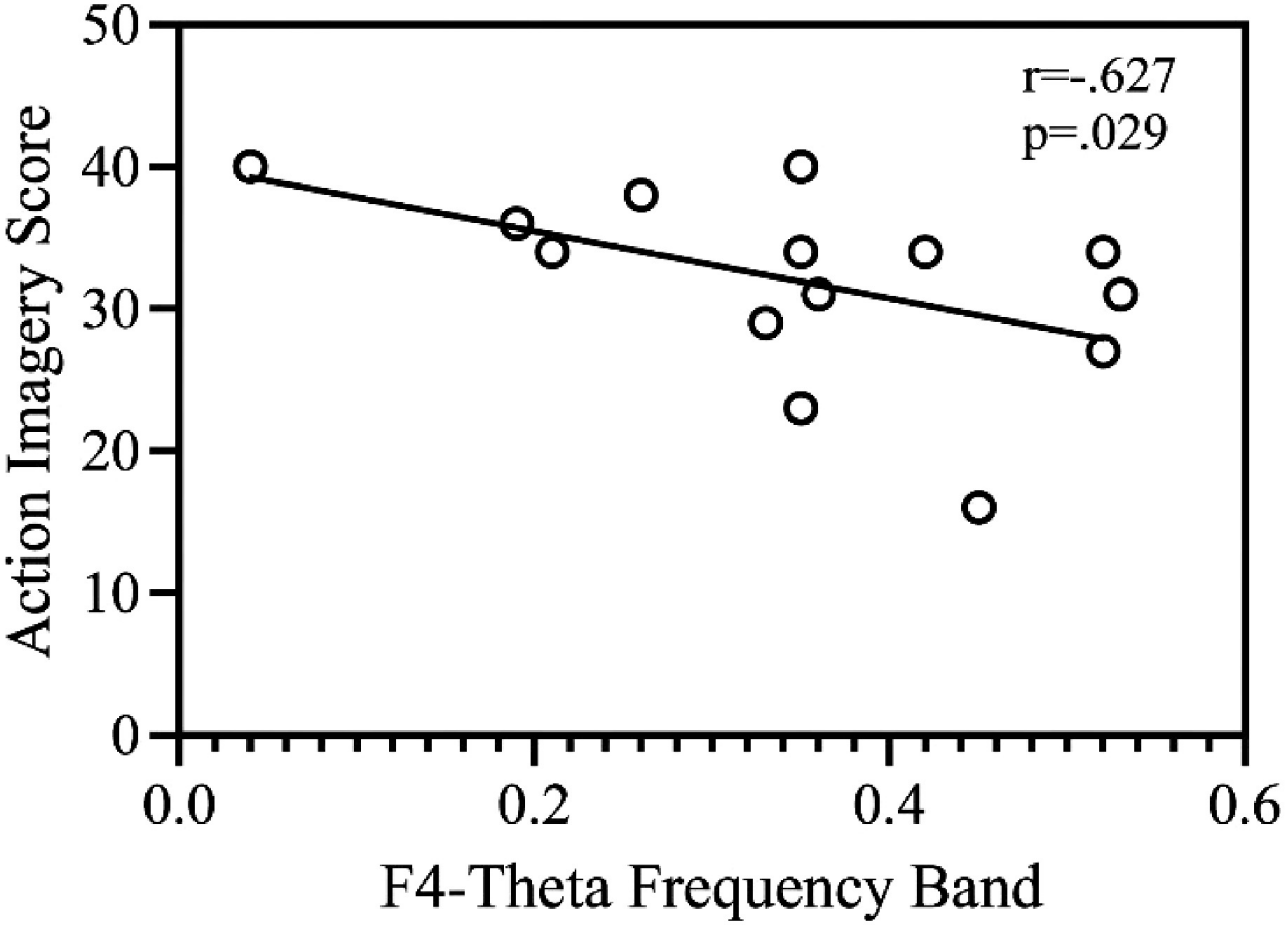
Correlation between the self-developed imagery self-rating total score and Fz-F4 theta-band coherence.

After FDR correction, the only significant association was observed under the high-difficulty imagery condition: right frontal Fz-F4 theta-band coherence was negatively correlated with the self-developed imagery self-rating total score, *r* = −0.627, *p* = 0.029, 95% CI [−0.880, −0.287] (Figure 6). No other correlations survived FDR correction.

## 4 Discussion

This study examined EEG coherence during the imagery phase of sport-specific action simulation in elite freestyle skiing aerials athletes, a context that is highly time-constrained, high-risk and dependent on body-space transformations. Behaviourally, high- and low-difficulty actions did not differ significantly in cue-video matching accuracy or imagery completion time, and mean accuracy in both conditions was only modestly above the nominal chance level. Thus, the behavioral data indicate that no difficulty-related behavioral difference was detected, but they should not be interpreted as evidence that high- and low-difficulty imagery involved equivalent processing. The EEG results nevertheless showed condition-related differences in sensor-level Fz coherence: high-difficulty actions primarily elicited stronger theta-band coherence between Fz and right-sided target electrodes; correct, compared with incorrect, judgements were associated with higher high-alpha coherence and with higher Fz-F4 and Fz-P4 theta coherence; and, in the high-difficulty condition, Fz-F4 theta coherence was negatively correlated with the self-developed imagery self-rating total score. These findings should therefore be interpreted as preliminary evidence that Fz-related EEG coherence may capture task-related processing not reflected in the behavioral measures, but they do not establish that the EEG effects reflect superior imagery quality. However, because behavioral accuracy was modest, the correct-versus-incorrect EEG contrasts should be interpreted cautiously. Instead, it may be reflected in changes in Fz-related EEG coherence during complex action simulation, posture prediction and cue-action comparison.

The theoretical contribution of the present study is that it treats sport-specific motor imagery in freestyle skiing aerials as an embodied predictive simulation process rather than as generic movement recall. In this process, athletes use dynamic in-run and take-off cues to construct an internal model of the subsequent aerial action, predict the unfolding body trajectory and compare this prediction with the cued action. Within this framework, theta-band coherence may reflect the control and updating demands required to organize a multistage aerial sequence, whereas high-alpha coherence may reflect the stabilization of the target imagery content and the inhibition of irrelevant or competing action representations. The absence of clear behavioral differences between high- and low-difficulty actions could also be interpreted as compatible with a neural-efficiency account, although neural efficiency was not directly measured: in elite athletes, long-term sport-specific experience may stabilize overt performance while task-dependent neural regulation remains visible in EEG coherence.

These results have important theoretical implications. Previous motor imagery studies have often used single-joint or simple limb movements, which are less able to capture the multistage internal simulation required by winter skill sports that involve continuous somersaults, twists and landing control. In the present task, athletes viewed external cues from the in-run to the moment of take-off, then internally completed the full aerial action and subsequently judged whether the cue and action matched. The current findings therefore do not reflect generic movement imagination. Rather, they characterize a process in which expert athletes transform external dynamic cues into an internal action model and then use the outcome of that model to support sport-specific judgment. Recent motor imagery work indicates that imagery and execution share part of their neural basis but that imagery, in the absence of overt motor feedback, additionally depends on internal prediction and control resources (Mustile et al., 2024). The present study extends this account to a sport-specific competitive context, showing that such internal predictive resources are expressed in theta- and alpha-band coherence, particularly in functional coupling between Fz and right-sided target electrodes. Recent fMRI and EEG studies of sport anticipation further indicate that experts integrate prior and kinematic information through expertise-sensitive neural dynamics and connectivity patterns, supporting the view that cue-based prediction is a central process in high-level sport cognition (Huang et al., 2026; Lu Y. et al., 2025; Wang C. et al., 2025).

### 4.1 Fz-right target-electrode theta coherence during high-difficulty imagery: complex spatial simulation and action prediction

Within the embodied predictive-simulation framework proposed here, the finding that high-difficulty actions elicited stronger theta-band coherence than low-difficulty actions suggests that complex aerial imagery required greater control and updating of the internal action model. This pattern is broadly consistent with the task demands of freestyle skiing aerials. High-difficulty actions typically contain more complex combinations of somersaults and twists. Athletes must not only maintain the temporal structure of the action sequence but also continuously update body orientation, center-of-mass position, take-off angle and potential landing posture. Enhanced theta coherence between Fz and right-sided target electrodes may therefore reflect more intensive internal integration of body-space relationships, rotation direction and postural transformation during high-difficulty imagery.

From a cognitive-neuroscience perspective, theta activity is often considered a key rhythm for prefrontal control, working-memory updating and the prioritization of internal representations. Sport-neurofeedback evidence also supports the relevance of frontal midline theta for precision sport performance, because function-specific neurofeedback instructions have been shown to influence frontal midline theta activity and golf putting performance (Chen et al., 2022a). Recent EEG-TMS and intracranial EEG evidence shows that frontoparietal theta connectivity helps prioritize task-relevant internal representations, whereas alpha dynamics participate in the suppression of irrelevant representations (Riddle et al., 2024). Consistent with this framework, recent work on the frontoparietal network during motor imagery has shown that transitions from rest to motor imagery are accompanied by task-related reconfiguration in the theta, alpha, beta and gamma bands, and that alpha/beta reconfiguration relates to better imagery classification performance (Chen et al., 2025). The increased theta coherence between Fz and right-sided target electrodes observed here can thus be interpreted as an Fz-related coordinated change that may support the prioritization and organization of sport-specific representations when high-difficulty aerial imagery places greater demands on the internal body model and spatial prediction. This interpretation is also consistent with basketball and broader sport expertise studies showing that skill level shapes the integration of contextual priors and kinematic cues, post-error adjustment and conflict processing during anticipation-related tasks (Chen et al., 2024; Gao et al., 2025). Badminton-specific evidence further suggests that expertise can improve early cue extraction and evidence accumulation when athletes make perceptual predictions from incomplete kinematic information (Wu et al., 2026).

The absence of behavioral differences in accuracy and imagery completion time does not imply equivalent processing load across high- and low-difficulty actions, but it also does not demonstrate that the observed coherence effects reflect superior imagery quality. In elite athletes, overt performance may become stabilized through long-term training, whereas EEG coherence may still capture task-related regulation associated with maintaining such stability. Recent evidence on motor expertise and imagery complexity suggests that experts may show lower cortical activation during imagery of simple actions but higher activation during imagery of complex actions, indicating that neural efficiency is not fixedly equivalent to reduced activation but is dynamically modulated by task complexity (Yin et al., 2025). On this basis, the stronger Fz-right target-electrode theta coherence elicited by high-difficulty actions may reflect the active recruitment of predictive and integrative resources during complex sport-specific simulation, but this interpretation remains preliminary and should not be taken as evidence of superior imagery quality.

### 4.2 Increased Fz-F4 and Fz-P4 theta coherence during correct judgments: exploratory candidate markers of successful cue-video matching

Fz-F4 and Fz-P4 theta coherence was higher during correct than incorrect judgments. This contrast should be interpreted cautiously because the behavioral accuracy was modest and the classification of trials into correct and incorrect outcomes may contain noise. Nevertheless, the contrast provides an exploratory way to examine whether imagery-phase coherence differed as a function of subsequent judgment outcome. Within this restricted interpretation, stronger Fz-F4 theta coherence may reflect greater task-goal maintenance, action-sequence monitoring or cue-action comparison during trials that were later judged correctly, whereas stronger Fz-P4 theta coherence may reflect coordinated changes between Fz-related control processes and right parietal sensor-level activity associated with body-space information processing.

This interpretation is consistent with contemporary neural oscillatory models of internal-representation prioritization. Riddle et al. (2024) showed that frontoparietal theta connectivity plays a key role in maintaining and selecting internal representations, whereas alpha oscillations help stabilize target representations by suppressing irrelevant content. Applied to the present task, stronger Fz-F4 and Fz-P4 theta coherence on correct trials may have reflected greater sensor-level coordination during imagery, but it should not be described as anatomically specific frontoparietal connectivity. Within this restricted interpretation, these Fz-related coherence changes may have allowed athletes to maintain the target action sequence more stably during imagery and to suppress competing action representations triggered by mismatched in-run or take-off information. In this sense, correct judgment may not arise only at the button-press stage; rather, it may already be supported during imagery by higher-quality coordinated changes across Fz-related electrode pairs.

### 4.3 Performance effects in high-alpha coherence: inhibition, stabilization and expert processing

Beyond the theta findings, high-alpha coherence was significantly higher during correct than incorrect judgments, whereas the performance effect in the low-alpha band did not reach statistical significance. This selective high-alpha effect is consistent with the rationale for separating alpha sub-bands, because upper-alpha activity has been more strongly associated with task-specific cognitive processing and controlled access to stored information than lower-alpha activity (Klimesch, 1999, 2012). Within the proposed framework, this alpha-band effect may reflect inhibitory stabilization rather than a simple increase or decrease in activation. In complex motor imagery tasks, alpha activity may serve two complementary functions: inhibiting visual or motor representations that are irrelevant to the current target action, and stabilizing an already formed internal action model so that it is not disrupted by external video cues or competing action plans. The increase in high-alpha coherence during correct judgments may therefore reflect a selective maintenance mechanism by which athletes preserved the clarity and consistency of the imagery outcome after completing the target action simulation.

This interpretation also helps explain the nonlinear characteristics of EEG functional coupling in expert athletes. EEG connectivity and expertise work in golfers has shown that functional connectivity and cognitive-motor coupling do not simply increase or decrease monotonically with skill level, but may show nonlinear refinement, reduced cognitive-motor coupling during successful difficult performance and psychomotor refinement in expert performance (Chen et al., 2022b; Wang et al., 2020, 2022, 2024). Accordingly, the present high-alpha coherence increase should not be interpreted as evidence that more activation is necessarily better, nor should it be directly generalized to source-space network conclusions. Rather, it should be understood as a form of functional organization specific to the task phase, frequency band and electrode combinations examined here. For freestyle skiing aerials, the key demand of the imagery phase is not to maximize cortical activation, but to form a stable action-outcome model within a limited time window that can subsequently support judgment. Increased high-alpha coherence may serve precisely this goal. More broadly, recent functional-connectivity work indicates that competitive sport experience is associated with distinct brain connectivity profiles beyond the immediate task context, suggesting that long-term sport experience may shape network organization relevant to action prediction and control (Huang et al., 2025).

### 4.4 Negative correlation between the self-developed imagery self-rating total score and Fz-F4 theta coherence: compensatory control or neural efficiency?

Exploratory correlation analysis showed that, during high-difficulty imagery, Fz-F4 theta coherence was negatively associated with the self-developed imagery self-rating total score after FDR correction. Because Fz-F4 theta coherence was selected on the basis of the ANOVA results and the sample size was small, this association should be interpreted cautiously and requires replication. At first glance, this pattern appears to conflict with the intuitive expectation that better imagery ability should be accompanied by stronger coherence. In expert athletes performing highly complex actions, however, this pattern may be cautiously interpreted as a possible manifestation of individual differences in neural-efficiency-like or compensatory-control processes. This cautious interpretation is consistent with sport-EEG evidence that higher skill can be associated with refined rather than globally stronger neural activity and with reduced cognitive-motor coupling in successful performance (Lu G. et al., 2025; Wang et al., 2020, 2021, 2022). Athletes with higher scores on the self-developed sport-specific imagery self-rating questionnaire may more readily access stable sport-specific action schemas and therefore may not need to rely excessively on control-related processing indexed by the Fz-F4 electrode pair during high-difficulty imagery. Conversely, athletes with lower subjective imagery ability may need to recruit additional control and monitoring resources to maintain the internal simulation of action sequences and spatial posture.

This interpretation does not deny the possibility of positive relationships between imagery ability and cortical activation. High-density EEG work has shown that gait imagery ability scores are associated with cortical activation in imagery-related networks, indicating that subjective imagery ability remains an important dimension for understanding the neural mechanisms of motor imagery (Putzolu et al., 2024). The key point is that the relationship between imagery ability and EEG coherence may be jointly moderated by task difficulty, sport expertise and measurement index. Consistent with recent findings in motor expertise, neural activity patterns during complex imagery in experts do not necessarily resemble those in novices and need not follow a single “higher ability-higher activation” model (Yin et al., 2025). The negative correlation observed here should therefore be interpreted cautiously as possible evidence of strategy differences within high-level athletes: some athletes may complete high-difficulty imagery with lower Fz-related control costs, whereas others may rely on stronger Fz-related compensatory processing to maintain imagery quality.

### 4.5 Limitations and future directions

Several limitations should be noted. First, the most important methodological limitation of the present study is that functional coupling was assessed using traditional magnitude-squared coherence at the sensor level. Although this approach is widely used and provides an intuitive index of frequency-specific synchronization between EEG channels, it is vulnerable to volume conduction, common reference effects and spatial leakage. These factors can artificially increase coherence between scalp electrodes, especially between relatively nearby electrode pairs such as Fz-F3 and Fz-F4. Therefore, the present findings should be interpreted as Fz-related sensor-level coherence signatures rather than as evidence of source-space connectivity or direct communication between cortical regions. This limitation is particularly important when interpreting the right frontal Fz-F4 theta effect. The effect may reflect task-related changes in functional coupling during successful sport-specific imagery, but it may also be partly influenced by spatial leakage or shared source activity. Future studies should test the robustness of these findings by applying methods that are less sensitive to zero-lag coupling, such as imaginary coherence, phase-lag index, weighted phase-lag index, surface Laplacian-transformed EEG or source-space connectivity analyses (Bastos and Schoffelen, 2016; Nolte et al., 2004; Stam et al., 2007; Vinck et al., 2011).

Second, the participants were members of the national training team, giving the sample high ecological value but also making it rare and small. The sample size of 14 limited statistical power and generalizability, and the sex distribution was imbalanced. Although FDR correction was applied to reduce the risk of Type I error arising from multiple EEG coherence tests, the small sample size means that the statistical findings should still be interpreted cautiously and should be replicated in larger samples. No a priori power analysis was conducted, and the sensitivity analysis showed that the available sample had adequate power only for large within-participant effects and large correlations. Accordingly, null findings should not be interpreted as evidence for the absence of an effect. The predominance of male athletes (12 men and 2 women) also prevents examination of sex-related differences and limits the generalizability of the findings to female freestyle skiing aerials athletes. Future studies should increase sample size through multi-center or multi-cohort data collection while maintaining control over competitive level.

Third, the laboratory video-cue paradigm used here is closer to a sport-specific context than traditional simple motor imagery tasks, but it still cannot fully reproduce on-snow training conditions, including speed, vestibular stimulation, muscle tension and risk pressure.

Fourth, although the correlation analysis revealed an association between Fz-F4 theta coherence and subjective imagery ability, the cross-sectional design cannot determine causal direction. Longitudinal imagery-training interventions are needed to test whether Fz-related theta and alpha coherence change with training and whether these indices predict real jump performance, action stability and landing success.

Fifth, the correlation analysis was exploratory because Fz-F4 and Fz-P4 theta coherence were selected after the ANOVA results. Although *p*-values were FDR-corrected across the 12 correlation tests and one association survived this correction, the small sample size means that the negative association between Fz-F4 theta coherence and the self-developed imagery self-rating total score should be interpreted cautiously. The cross-sectional design also cannot determine causal direction. Longitudinal imagery-training interventions are needed to test whether Fz-related theta and alpha coherence change with training and whether these indices predict real jump performance, action stability and landing success.

Finally, a further limitation concerns the subjective assessment of imagery. Because the present task involved highly sport-specific cue-video matching and aerial action imagery, we used self-developed task-specific questionnaires rather than an externally validated general motor imagery scale. Although the internal consistency was high in the present cohort for both the Video Attention and Action Focus Questionnaire and the Sport-Specific Motor Imagery Self-Rating Questionnaire, Cronbach’s alpha provides evidence of internal consistency only and does not by itself establish construct validity (Tavakol and Dennick, 2011). No independent content-validity index, factor-structure analysis, test-retest reliability, convergent validity or criterion validity assessment was conducted. Therefore, the questionnaire scores should be regarded as preliminary task-specific self-report indices rather than fully validated psychometric measures. Future studies should further validate these questionnaires in larger athlete samples and follow scale-development recommendations by examining content validity, dimensionality, reliability, and construct and criterion validity (Boateng et al., 2018).

## 5 Conclusion

Sport-specific motor imagery in freestyle skiing aerials athletes is not merely a recall of movement, but may constitute an embodied predictive simulation process. In this process, athletes use in-run and take-off cues, together with accumulated sport-specific sensorimotor experience, to predict, organize and refine the subsequent aerial action. The present findings show condition-related differences in sensor-level Fz coherence during high-difficulty imagery and successful judgments. In particular, Fz-F4 and Fz-P4 theta coherence and high-alpha coherence should be viewed as preliminary sensor-level EEG coherence signatures rather than established neural markers of imagery quality or successful sport-specific judgment. These conclusions should be considered preliminary because behavioral cue-video matching accuracy was modest, the correlation analyses were exploratory and FDR-corrected, and the correct-versus-incorrect EEG contrasts require replication with stronger behavioral performance and larger samples.

## Data Availability

The original contributions presented in this study are included in this article/supplementary material, further inquiries can be directed to the corresponding author.
